# Manic episode secondary to single right cerebellar metastasis in context of ovarian tumor: report of clinical case

**DOI:** 10.1192/j.eurpsy.2022.562

**Published:** 2022-09-01

**Authors:** A. Téllez Gómez, M.T. Rubio Granero

**Affiliations:** Hospital Universitario y Politécnico La Fe, Department Of Psychiatry And Psychology, Valencia, Spain

**Keywords:** cognitive affective cerebellar syndrome, metastasis, mania, cerebellum

## Abstract

**Introduction:**

The cerebellum is often overlooked when evaluating neuropsychiatric disorders. Lately, evidence has increased for the existence of cerebellar connections -generally in relation to vermis and posterior lobe- with cortical areas related to pathophysiology of psychiatric disorders. The cerebellar affective cognitive syndrome, also known as Schmahmann syndrome, has even been described with an evaluation scale.

**Objectives:**

Case report of a patient suffering a manic episode in context of single right cerebellar metastasis derived from ovarian tumor.

**Methods:**

A non-systematic literature review was conducted on PubMed database on cerebellum pathology related to psychiatrics disorders. The clinical case report was prepared through the review of the patient´s clinical record.

**Results:**

The authors introduce the case of a 50-year-old woman, diagnosed with high-grade serous ovarian tumor, with single right cerebellar metastasis of 42mm, who was admitted to oncology due to behavioral alteration, with no prior psychiatric history. The patient showed hyperthymic mood, with speech scanned but fluid, manifesting intense well-being and ideation of mystical-religious and megalomaniac content. Haloperidol up to 7.5mg/8 hours and clonazepam 2 mg/8 hours were prescribed, switching haloperidol to olanzapine up to 25mg/day after several days, since the symptoms did not improve. Valproic acid 500 mg/24 hours was also added. Progressive improvement was seen, without worsening of motor symptoms or instability. The CCAS scale (Cerebellar Cognitive Affective/Schmahmann syndrome) was performed, with a positive result (10/10) being> 3 definitive CCAS.

**Conclusions:**

The relationship between cerebellum and neuropsychiatric disorders is still partly unknown, requiring more research to be able to carry out specific diagnoses and treatments for patients.

**Disclosure:**

No significant relationships.

